# RE: Sheehan syndrome with reversible dilated cardiomyopaty

**DOI:** 10.4103/0256-4947.72278

**Published:** 2010

**Authors:** Sourabh Aggarwal

**Affiliations:** University College of Medical Sciences, New Delhi, India drsourabh79@gmail.com

**To the Editor**: I read the case report on ‘Sheehan syndrome with reversible dilated cardiomyopaty’^1^ by Laway et al with interest. The case indeed looks interesting. However, it could also have been a case of peripartum cardiomyopathy, which can have an onset up to 5 months after pregnancy^2^ and in most cases, recovery of left ventricular function occurs within 6 months of its diagnosis.^2^,^3^ Also, a previous history of subclinical hypothyroidism cannot be ruled out, which might have been precipitated by the peripartum events. The authors also failed to mention the details of antitubercular therapy, which could have altered the course of disease and contributed to drug-induced dilated cardioyopathy.
